# Fractal analysis of ^11^C-methionine PET in patients with newly diagnosed glioma

**DOI:** 10.1186/s40658-021-00418-y

**Published:** 2021-11-06

**Authors:** Yukito Maeda, Yuka Yamamoto, Takashi Norikane, Katsuya Mitamura, Tetsuhiro Hatakeyama, Keisuke Miyake, Yoshihiro Nishiyama, Nobuyuki Kudomi

**Affiliations:** 1 Department of Clinical Radiology, Kagawa University Hospital, 1750-1 Ikenobe, Miki-cho, Kita-gun, Kagawa 761-0793 Japan; 2 Department of Radiology, Faculty of Medicine, Kagawa University, Miki-cho, Kagawa Japan; 3 Department of Neurological Surgery, Faculty of Medicine, Kagawa University, Miki-cho, Kagawa Japan; 4 Department of Medical Physics, Faculty of Medicine, Kagawa University, Miki-cho, Kagawa Japan

**Keywords:** Fractal analysis, Glioma, PET, ^11^C-methionine, Grading, IDH1

## Abstract

**Background:**

The present study tested the possible utility of fractal analysis from l-[methyl-^11^C]-methionine (MET) uptake in patients with newly diagnosed gliomas for differentiating glioma, especially in relation to isocitrate dehydrogenase 1 (IDH1) mutation status, and as compared with the conventional standardized uptake value (SUV) parameters.

**Methods:**

Investigations of MET PET/CT were performed retrospectively in 47 patients with newly diagnosed glioma. Tumors were divided into three groups: lower grade glioma (IDH1-mutant diffuse astrocytoma and IDH1-mutant anaplastic astrocytoma), higher grade glioma (IDH1-wildtype diffuse astrocytoma and IDH1-wildtype anaplastic astrocytoma), and glioblastoma. The fractal dimension for tumor, maximum SUV (SUVmax) for tumor (*T*) and mean SUV for normal contralateral hemisphere (*N*) were calculated, and the tumor-to-normal (*T*/*N*) ratio was determined. Metabolic tumor volume (MTV) and total lesion MET uptake (TLMU) were also measured.

**Results:**

There were significant differences in SUVmax (*p* = 0.006) and *T*/*N* ratio (*p* = 0.02) between lower grade glioma and glioblastoma. There were no significant differences among any of the three groups in MTV or TLMU. Significant differences were obtained in the fractal dimension between lower grade glioma and higher grade glioma (*p* = 0.006) and glioblastoma (*p* < 0.001).

**Conclusions:**

The results of this preliminary study in a small patient population suggest that the fractal dimension using MET PET in patients with newly diagnosed gliomas is useful for differentiating glioma, especially in relation to IDH1 mutation status, which has not been possible with SUV parameters.

## Background

Accurate histopathological grading of brain gliomas is important for devising the treatment plan and predicting prognosis [1]. In addition, isocitrate dehydrogenase (IDH) mutation is the genetic alteration with the most significant impact noted in the updated 2016 edition of the World Health Organization (WHO) classification of tumors of the central nervous system [2]. Because tissue sampling is often obtained by stereotactic biopsy and, therefore, represents only a small part of the tumor, the true tumor grade is likely to be underestimated [3]. Thus, noninvasive imaging-based technique to detect malignant progression is required to select the best possible treatment regimen.

Positron emission tomography (PET) with l-[methyl-^11^C]-methionine (MET) has been widely used as a brain imaging tool for tumor detection, tumor grading and prediction of prognosis in patients with gliomas [4]. Furthermore, recent reports have documented that MET uptake in IDH1-wildtype gliomas is significantly higher compared with that in IDH1-mutant gliomas [5]. To date, the most commonly used PET parameters are the standardized uptake value (SUV) derived indices. Previously, our research team reported that MET SUV parameters were useful for differentiation between grades II and IV gliomas but not between grades II and III gliomas or grades III and IV gliomas [6]. Beyond the relatively simple measurements of the level of tumor uptake, recently, texture and fractal analyses have attracted attention as semiquantitative methods. Fractals were introduced by Mandelbrot to characterize structures and processes occurring in nature [7]. Miwa et al. demonstrated that fractal analysis using 2-deoxy-2-[^18^F]fluoro-d-glucose (FDG) PET is useful for discriminating benign from malignant pulmonary nodules [8]. Nakajima and colleagues evaluated modified fractal analysis of MET PET for predicting prognosis in patients with newly diagnosed gliomas [9]. In their study, modified fractal dimension was significantly associated with a poor prognosis [9]. However, the experience with PET fractal analysis is still limited.

The purpose of the present study was to test the possible utility of fractal analysis from MET uptake in patients with newly diagnosed gliomas for differentiating glioma, especially in relation to IDH1 mutation status, in a comparison with the conventional SUV parameters.

## Methods

### Patients

This retrospective study was approved by our institutional ethics committee with the need for obtaining informed consent waived.

Complete data on MET PET/CT before therapy and patients with newly diagnosed gliomas that were classified or reclassified using the 2016 WHO classification were available for 54 patients from May 2010 to June 2017. Of them, oligodendroglial tumors were excluded because they appreciably affect the results of MET PET due to their high blood volume and high blood flow within tumor. Finally, 47 patients (22 men, 25 women; mean age, 61.6 years; age range 21–86 years) were enrolled in the study. Histopathology including immunohistochemistry (IHC) was performed on tissue specimens obtained by biopsy or resection. All gliomas were classified or reclassified using the 2016 WHO classification. The presence of IDH1 mutation was assessed by IHC to detect IDH1 R132H (codon 132 of the IDH1 gene) protein expression. IDH1 sequencing was performed when the IHC studies were negative. Their clinical characteristics are summarized in Table 1. Tumors were divided into three groups: lower grade glioma, higher grade glioma, and glioblastoma. The "lower grade glioma" comprised both IDH1-mutant diffuse astrocytoma and IDH1-mutant anaplastic astrocytoma. The "higher grade glioma" comprised both IDH1-wildtype diffuse astrocytoma and IDH1-wildtype anaplastic astrocytoma.

**Table 1:** Patient clinical characteristics

Characteristic		Value
Age (years)	Mean	61.6
	Range	21–86
Sex (n)	Male	22
	Female	25
Histology (n)	IDH1 mutation	
Diffuse astrocytoma	Mutant	3
	Wildtype	1
Anaplastic astrocytoma	Mutant	3
	Wildtype	6
Glioblastoma	Mutant	3
	Wildtype	31

### MET synthesis and PET/CT

The MET was synthesized using a modified method of Ishiwata et al. [10].

All acquisitions were performed using a Biograph mCT 64-4R PET/CT scanner (Siemens Medical Solutions USA Inc., Knoxville, TN, USA). PET emission scanning of the head region with a 10-min acquisition of one bed position was performed 20 min after intravenous injection of MET (6 MBq/kg). The PET data were reconstructed with the ordered-subsets expectation maximization algorithm with time-of-flight information. The reconstruction parameters were 4 iterations and 21 subsets. A Gaussian filter with a full width at half maximum of 4 mm was used. The image matrix generated was 256 × 256, with 1.26-mm pixels, and the slice thickness 3 mm. A whole-brain CT scan protocol using the following parameters was performed: 120 kV, 50 mA, 0.5-s tube rotation, and 3-mm slice collimation. CT data were used for attenuation correction.

### Data analysis

The volume of interest (VOI) of the tumor on PET images was selected using a threshold of 40% of the maximum SUV (SUVmax). For the reference tissue, a circular region of interest (ROI) of 10 × 10 mm was placed manually on the uninvolved contralateral hemisphere. The tumor–to–contralateral normal brain tissue (*T*/*N*) ratio was determined by dividing the tumor SUVmax by the mean SUV (SUVmean) of the reference tissue [11]. Metabolic tumor volume (MTV) was derived from the same VOI. Total lesion MET uptake (TLMU) was calculated as follows: MTV × SUVmean for tumor.

The Custom Fractal Version 1.0 (Digital being kids Ltd, Tokyo, Japan) was used to measure the fractal dimension. VOI with the minimum size required to cover the entire tumor and the slice with maximum ROI diameter was set manually. From the extracted pixel value, we measured the fractal dimension using the pixel counting method as follows. First, we extracted the mean values from the obtained pixel values. We set a width that would contain 20 steps between the minimum and mean values. Threshold values [*T* (Bq/mL)] were set as cut-offs determined from the minimum to maximum pixel values. Then, the number of pixels above the threshold was defined as the pixel count *M*(*T*). The fractal dimension (*D*) can be estimated by the following formula [12]:

$$M\left( \varepsilon \right) = k \cdot \varepsilon^{ - D} ,$$

where *k* is a constant which takes into account the number of pixels extracted from tumor regions and *ε* indicates a scale which is, in the present estimation, given by the threshold *T* and the fractal dimension can be estimated as a slope of the linear relation when the formula is expressed in log scale as:

$$\ln M\left( \varepsilon \right) = \ln k - D\ln \varepsilon .$$

Finally, ln(*M*) was plotted against *T*, and *D* was obtained using the linear least square method, with the range adapted for each patient (Table 2).

**Table 2:** Threshold range for determining the fractal dimension in patients with newly diagnosed gliomas

Glioma group	Patient no.	Activity concentration (kBq/mL)	
		Lower limit	Upper limit
Lower grade glioma			
	1	8.9	13.8
	2	8.8	11.6
	3	7.0	10.2
	4	9.0	12.3
	5	7.4	12.8
	6	6.8	9.8
Higher grade glioma			
	7	10.7	14.4
	8	9.7	14.6
	9	11.2	20.1
	10	8.4	15.5
	11	5.6	6.9
	12	8.7	14.6
	13	5.6	10.2
Glioblastoma			
	14	9.4	18.2
	15	9.4	17.3
	16	6.3	9.2
	17	9.1	16.5
	18	11.2	21.8
	19	12.0	23.4
	20	8.1	13.3
	21	6.4	11.6
	22	7.3	11.2
	23	6.6	11.0
	24	12.7	24.5
	25	11.9	21.6
	26	7.4	15.1
	27	10.6	20.8
	28	9.0	13.0
	29	10.2	24.0
	30	8.9	13.0
	31	11.4	24.0
	32	8.0	12.5
	33	8.8	19.4
	34	13.2	24.7
	35	7.5	15.2
	36	12.1	19.5
	37	11.4	21.4
	38	7.3	11.1
	39	5.9	10.5
	40	8.2	15.3
	41	10.1	17.3
	42	10.1	16.7
	43	8.2	16.8
	44	10.1	20.5
	45	7.3	13.2
	46	9.4	17.7
	47	6.1	10.0

### Statistical analysis

All parametric data were expressed as mean ± SD. Differences in semiquantitative data among glioma groups were compared using analysis of variance and post hoc comparisons with Bonferroni correction. All data were statistically analyzed using SPSS software (SPSS Inc., USA, Ver. 26), and *p* < 0.05 was considered to indicate statistical significance.

## Results

**Fig. 1:**
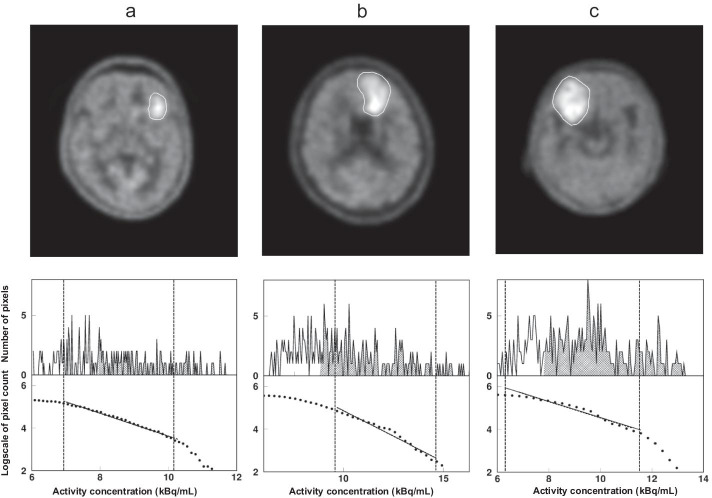
PET images (upper panel) for lower grade glioma (IDH1-mutant diffuse astrocytoma) (patient no. 3 in Table 2) (**a**), higher grade glioma (IDH1-wildtype anaplastic astrocytoma) (patient no. 8 in Table 2) (**b**), and glioblastoma (patient no. 21 in Table 2) (**c**) and corresponding histograms for pixel count and logscale pixel number above the threshold as a function of pixel value (kBq/mL) in the tumor area (lower panel). The shaded area represents the numbers of pixels above thresholds, and the obtained number are plotted as the pixel number. The dashed line shows upper and lower limits for fractal analysis. The slope of the line on the points plotted indicates the fractal dimension

Table 3 summarizes the results of the MET PET parameters. Figure 1 shows typical cases of lower grade glioma, higher grade glioma, and glioblastoma. Significant differences were noted in SUVmax (*p* = 0.006) and *T*/*N* ratio (*p* = 0.02) between lower grade glioma and glioblastoma. No significant differences in SUV or *T*/*N* ratio were noted between lower grade glioma and higher grade glioma (*p* = 0.32 and *p* = 0.55, respectively) or higher grade glioma and glioblastoma (*p* = 0.56 and *p* = 0.53, respectively). No significant differences in MTV or TLMU were noted between lower grade glioma and higher grade glioma (*p* = 0.26 and *p* = 0.57, respectively) or lower grade glioma and glioblastoma (*p* = 0.15 and *p* = 0.09, respectively) or higher grade glioma and glioblastoma (*p* = 1.00 and *p* = 1.00, respectively).

**Table 3:** MET PET findings in patients with newly diagnosed gliomas according to the glioma group

MET PET	Lower grade glioma (*n* = 6)		Higher grade glioma (*n* = 7)		Glioblastoma (*n* = 34)	
Parameter	Mean	SD	Mean	SD	Mean	SD
SUVmax	4.17	0.78	5.91	2.02	6.96	1.99
*T*/*N* ratio	2.95	1.75	4.08	1.93	4.94	1.49
MTV	3.28	2.91	15.05	22.26	14.07	9.99
TLMU	10.03	8.81	51.06	73.52	65.38	55.58
FD	0.000336	0.000114	0.000162	0.000067	0.000158	0.000095

*SUVmax* maximum standardized uptake value, *MTV* metabolic tumor volume, *TLMU* total lesion MET uptake, *FD* fractal dimension

Significant differences were found in the fractal dimension between lower grade glioma and higher grade glioma (*p* = 0.006) and glioblastoma (*p* < 0.001). There was no significant difference in the fractal dimension between higher grade glioma and glioblastoma (*p* = 1.00).

## Discussion

In this study, we tested the possible utility of fractal dimension from MET uptake in patients with newly diagnosed gliomas for differentiating glioma, especially in relation to IDH1 mutation status. The fractal dimension seemed to be useful, especially for differentiating IDH1 mutation status, which has not been possible with SUV parameters.

In the present study, MET SUVmax and *T*/*N* ratio in glioblastoma were significantly higher than those in IDH1-mutant diffuse astrocytoma and anaplastic astrocytoma. MET PET seemed to be useful, but not sufficiently so. MET SUVmax and *T*/*N* ratio did not allow differentiation between IDH1-mutant and IDH1-wildtype in diffuse astrocytoma and anaplastic astrocytoma or IDH1-wildtype diffuse astrocytoma and anaplastic astrocytoma and glioblastoma. In the previous reports focused on the glioma grade, although MET SUVmax and *T*/*N* ratio could differentiate between grades II and IV gliomas, they did not between grades II and III gliomas or III and IV gliomas [6, 13, 14]. On the other hand, the present MET fractal dimension showed a significant difference between IDH1-mutant and IDH1-wildtype in diffuse astrocytoma and anaplastic astrocytoma and IDH1-mutant diffuse astrocytoma and anaplastic astrocytoma and glioblastoma. The differentiation between IDH1-mutant and IDH1-wildtype is extremely important, because both the response to and benefit of treatment differ depending on the IHD1 mutation status [15, 16]. However, in this study, similar to SUV analysis, even fractal analysis was unable to distinguish between IDH1-wildtype diffuse astrocytoma and anaplastic astrocytoma and glioblastoma. To date, only the study of Nakajima et al. has published results using fractal analysis of MET PET according to the glioma grade [9]. They observed no significant differences between the fractal dimension and gliomas of any grade [9]. One of the reasons for this discrepancy may be differences in the histological types of gliomas. In their cases, there were relatively high oligodendroglial tumor components. Increased MET uptake was observed not only in pure oligodendroglioma but also in mixed oligoastrocytoma [14]. Therefore, we excluded oligodendroglial tumors from the present study. Further research with larger patient populations will be needed to determine the reliability of fractal analysis for glioma differentiation based on the 2016 WHO classification.

Miwa et al. evaluated lung nodules using FDG fractal analysis, and documented that the density fractal dimension of malignant nodules was significantly lower than that of benign nodules [8]. Similarly, we found that the higher the degree of malignancy of gliomas, the lower was the density fractal dimension. However, the ranges of fractal dimensions were quite wide which made it difficult to establish proper thresholds for clinical use. The setting of the tumor's area and its size may have accounted for the wide range of fractal dimensions. As we noted in the methods, we changed stepwise the threshold setting and plotted the number of pixels above the threshold as a function of pixel value. The plot was linearly fitted and the slope of the line represented the fractal dimension. The fractal dimension increased when the number of pixels decreased rapidly like when the threshold increased, and decreased when the number decreased slowly. In other words, the fractal dimension is considered to measure the pattern of the radioactivity concentration distribution in the ROI. In this study, an adaptive threshold, instead of a fixed threshold, was used for each patient. Yet such an adaptive threshold method might be less reproducible. It might have been better to set a threshold for each group of patients, since repeatability and reproducibility are fundamental requirements for quantitative assessment. The setting of thresholds for determining the fractal dimension has not been established yet. For an optimal range, a cohort study with a large number of subjects would be needed.

Limitations of the present study include its small sample size and retrospective design. Especially, it included only 4 diffuse astrocytoma patients. In our institution, brain tumors expected to be diffuse astrocytoma using diagnostic imaging do not immediately proceed to surgical pathological evaluation, and instead their progression is observed. To resolve these issues, multicenter trials aiming to collect many cases are warranted. Methods for fractal analysis have not been established. As mentioned above, the range for fractal dimension estimation was set, but has not yet addressed smaller subjects or optimization-related issues. Reproducibility was not validated here, despite its importance for quantitative evaluation. This study investigated the possibility of applying fractal dimension for differentiating glioma but did not evaluate diagnostic performance by parameters such as receiver-operating-characteristic curves, sensitivity, and specificity. In the future, we will compare the fractal dimension and other semiquantitative parameters such as SUV to evaluate diagnostic performance.

## Conclusion

The results of this preliminary study albeit from a small patient population suggest that the fractal dimension using MET PET in patients with newly diagnosed gliomas is useful for differentiating glioma, especially in relation to IDH1 mutation status, which has not been possible with SUV parameters.

## Data Availability

The data that support the findings of this study are available from the corresponding author on reasonable request.

## Abbreviations

PET: Positron emission tomography
MET: l-[Methyl-^11^C]-methionine
SUV: Standardized uptake value
FDG: 2-Deoxy-2-[^18^F]fluoro-d-glucose
ROI: Region of interest
SUVmax: Maximum SUV
SUVmean: Mean SUV
*T*/*N*: Tumor-to-contralateral normal brain tissue
